# Fabrication and characterization of polycaprolactone/cellulose acetate blended nanofiber mats containing sericin and fibroin for biomedical application

**DOI:** 10.1038/s41598-022-26908-2

**Published:** 2022-12-26

**Authors:** Naris Barnthip, Jantima Teeka, Puripat Kantha, Siriwan Teepoo, Watchara Damjuti

**Affiliations:** 1 Division of Physics, Faculty of Science and Technology, Rajamangala University of Technology Thanyaburi, Pathum Thani, 12110 Thailand; 2 Division of Biology, Faculty of Science and Technology, Rajamangala University of Technology Thanyaburi, Pathum Thani, 12110 Thailand; 3 Department of Chemistry, Faculty of Science and Technology, Rajamangala University of Technology Thanyaburi, Pathum Thani, 12110 Thailand; 4 Department of Integrative Medicine, Faculty of Integrative Medicine, Rajamangala University of Technology Thanyaburi, Pathum Thani, 12130 Thailand

**Keywords:** Biomedical materials, Nanobiotechnology, Nanoscale materials

## Abstract

Polycaprolactone/cellulose acetate blended nanofiber mats containing sericin and fibroin were fabricated by electrospinning process to study the effect of sericin and fibroin on the physical and structural properties, wettability, degradability, elastic modulus, cell adhesion, and cell cytotoxicity of the electrospun nanofibers. Polycaprolactone/cellulose acetate solution was prepared with different percentage ratio of sericin and fibroin to be the running solution. Nanofibers were spun at fixed solution flow rate, flying distance, and operating voltage. The diameter of the obtained nanofibers linearly increases with the increasing of the sericin ratio. The derivative structures of polycaprolactone, cellulose acetate, sericin, and fibroin of the obtained nanofibers were confirmed by FTIR analysis. All acquired nanofibers show superhydrophilicity with adequate time of degradation for L-929 cell adhesion and growth. More elasticity is gained when the sericin ratio decreases. Moreover, all fibers containing sericin/fibroin reveal more elasticity, cell adhesion, and cell growth than that with only polycaprolactone/cellulose acetate. Greater cell adhesion and growth develop when the sericin ratio is lower. All the fabricated nanofibers are low toxic to the cells. Fibers with a mixture of sericin and fibroin at 2.5:2.5 (% w/v) are the most promising and suitable for further clinical development due to their good results in each examination. The novelty found in this study is not only making more value of the sericin, silk industrial waste, and the fibroin, but also getting the preferable biomaterials, scaffold prototype, with much greater mechanical property and slower degradation, which are required and appropriate for cell attachment and proliferation of cell generation process, compared to that obtaining from polycaprolactone/cellulose acetate or sericin/fibroin nanofibers.

## Introduction

It is undeniable today that the rate of deterioration of the human body is increasing rapidly. This can be seen from the morbidity rates of various diseases or injuries from accidents which lead to the deterioration of the body. With increasing recent technology in medical science, damaged or diseased cells can be repaired or replaced by culturing cells in scaffolds^1–5^. This method known as tissue engineering combines the principles of engineering and biology. Tissue engineering is being studied more and more in many countries continuously to reduce the import of medical products and technologies from abroad.

There are many researches that can develop and create bio-substitute materials for cell regeneration called scaffolds which allow cells to adhere, multiply, and regenerate into complete tissues^6–8^. Biomaterials used as scaffolds should have initial properties that can promote cell function with no defense response from the human body, and be able to naturally degrade. Scaffolds that are commonly invented and used today are often in the form of nanometer-scale fibers (nanofibers) because the nanofibers possess a high surface-to-volume ratio, resulting in the movement and proliferation of cells rapidly in the process of tissue formation^9–11^. Nanofibers are also found to be applied for blood vessel transplantation process, wound dressing, and drug delivery systems^12–18^.

A popular process for fabricating nanofiber sheets today is electrostatic spinning (electrospinning process) because it is a simple, uncomplicated, and efficient process^19–21^. Both synthetic and natural polymers which can be used in medicine such as polyvinyl alcohol, polylactic acid, polycaprolactone, chitin, chitosan, cellulose, collagen, keratin, and silk have been studied and fabricated into nanofiber sheets. These polymers are biocompatible, compatible with living cells, with no toxicity with and remains in the body^22–24^.

Polycaprolactone (PCL) is a biodegradable polymer that is gaining attention as a replacement for polylactic acid (PLA), which has been used in early medical applications. PCL has a structure like PLA, but it exhibits a slower degradation. The PCL degrades in 24 months, while the PLA degrades in only 4 to 5 months^25,26^.

Cellulose acetate (CA) is a cellulose derivative. It is a biodegradable and easy to form polymer. CA is widely used for selective membranes and materials in medical applications due to its good stability, great compatibility with living systems, and low cost^27,28^.

Many kinds of nature materials such as cellulose, rubber, collagen, and silk are used for different biomedical applications^29–32^. Bombyx Mori silk consists of two main proteins, fibroin (SF) and sericin (SS), which are fibers that are joined together^31,32^. SS is considered as the silk industrial waste during reeling process. Medical property of SS is reported widely for its antioxidant and anti-inflammatory. In addition, SS helps to enhance the cell adhesion and mechanical property, and to inhibit the bacterial growth^33^. SF which is classified as a biological substance used in the production of cell scaffolds shows an ability to induce low-level resistance and response from the human immune system, resulting in great compatibility with biological systems. Moreover, it also naturally and slowly degrades, and helps to increase in cell adhesion, resulting in better cell growth^31^. SF was also used for other biomedical applications such as drug combination treatment in the form of microneedle patch due to its biocompatibility and biodegradability helping in sustained release of multiple drugs at different doses^34^. Numerous studies have been conducted on the fabrication of nanofibers from different polymers and SS mixtures, and several studies have also been conducted on the fabrication of nanofibers from CA containing SF. However, very few studies have been investigated on the fabrication of SS/SF nanofibers or the nanofibers from a polymer solution containing both SS and SF.

The desired scaffolds are required to have the mechanical properties close to the native tissue or organ^35^. Cortical bone and cancellous bone are reported to have the Young’s modulus of 7–30 GPa and 0.05–0.5 GPa, sequentially^36^ while the elastic modulus of the PCL nanofibers and SS/SF nanofibers are only at 60 MPa^37^ and 5.5 MPa^38^, respectively. Moreover, cell attachment and proliferation are usually determined at 1–3 days of cell culture with preferred scaffolds^39^. It is also reported that the cell regeneration is last for several weeks^36^. However, the SS/SF nanofiber mat is easily dissolved in water within 1 h^40^, which is clearly seen that it is not appropriate for being as a scaffold for cell culture and regeneration. Therefore, greater mechanical and biomedical properties, and high elasticity with slow degradation of the fabricated materials containing the combination of SS and SF will be expected for solving the mentioned issues.

By the study of the properties of the polymers and nanofiber sheets as mentioned above, this led researchers to be interested in fabricating PCL/CA nanofibers containing SS and SF by electrostatic spinning process to be applied as a scaffold for damaged tissues in different areas of the body. This study aims to study the influence of different proportions of SS and SF on the properties of nanofibers including the possibility or potentiality to develop an extended scaffold prototype that will work and great for medical application in the future. Physical and surface characteristics, molecular structural property, wetting property, biodegradable property, tensile strength, cell adhesion and formation, and cell cytotoxicity are performed.

## Materials and method

### Nanofiber fabrication

Polycaprolactone powder (PCL), cellulose acetate powder (CA), and trifluoroacetic acid (TFA) were purchased from Sigma-Aldrich. Silk sericin (SS) and silk fibroin (SF) powder were purchased from Quanao Biotech Co., Ltd. (China). All chemicals were used as received without further purification. Specific amount of PCL and CA powder were dissolved in TFA at room temperature and mixed with magnetic stirrer to obtain 15% (w/v) CA and 1% (w/v) PCL solution. SS and SF were added into the PCL/CA solution with 7 different percentage ratios of 0.0:0.0, 2.5:2.5, 3.0:2.0, 3.5:1.5, 4.0:1.0, 4.5:0.5, and 5.0:0.0 (w/v) in total solution and mixed with magnetic stirrer at 37 °C for 4 h. Nanofiber mats were fabricated with the prepared solution by electrospinning technique adapted from previous works^41–44^ at fixed experimental operating voltage, flow rate, distance between the needle tip and collector, and time of 15 kV, 0.3 mL/h., 10 cm., and 2 h., respectively. The collector was covered by the aluminum foil before fabricating the fibers. Obtained fibers were dried in an electric oven at 37 °C for 48 h. and kept in a desiccator for further characterization.

### Morphological structures

Fabricated nanofibers obtained from all experimental conditions were observed for their morphological structures by a scanning electron microscope (SEM, JSM-5410LV, JEOL, Japan). The fiber size was analyzed by ImageJ software. The average fiber size and standard derivation were calculated based on 10 different locations of each fiber for at least 10 fibers of each experimental condition.

### Molecular structural property

The composition of the fabricated nanofibers was roughly investigated from the characteristic pattern of adsorption bands from the Fourier-transform infrared spectroscopy analysis (FTIR, Nicolet iS5, Thermo Scientific, USA). PCL, CA, SS, and SF powders were also identified with FTIR technique for their spectrum used as the reference composition.

### Wetting property

The wettability of the fabricated fibers was determined by the contact angle between the water droplet and the fiber mat surface which was evaluated by a contact angle meter (DM-CE2, Kyowa, Japan) via the sessile drop method. The average contact angle and standard derivation were calculated based on 10 random locations of each fiber mat from each experimental condition.

### Biodegradable property

Biodegradation of obtained nanofiber mats was analyzed by immersion method. The fibers were immersed in a simulated body fluid (SBF) solution, which is favorably used as a standard solution for assessing materials bioactivity^45^, at a pH of 7.4 and at a temperature of 37 °C to observe the mass change of the fiber mats. The initial dried mass of the fiber mats (M_i_) from each condition was balanced before immersion. The dried mass of the fiber mats after immersion (M) at 1, 3, 5, 7, 9, 11, 13 and 15 days were observed. Persistence and degradation percentages of the fiber mats were calculated from Eqs. (1) and (2), respectively.

1 $$\% \; {\text{Persistence}} = \left[ {{\text{M}}/{\text{M}}_{{\text{i}}} } \right] \times {1}00$$

2 $$\% \; {\text{Degradation}} = {1}00 - \% \; {\text{Persistence}}$$

### Tensile strength

Tensile strength of the fabricated nanofibers was characterized by the tensile testing machine (5569, Instron, USA) with the pulling force and speed of 10 N. and 10 mm./min., respectively. Young’s Modulus of elasticity of each fiber mat was calculated based on the stress and strain interpretation.

### Cell adhesion and formation

Cell adhesion and formation on the fabricated nanofiber mats were performed in 96-well plate. The nanofibers were pre-sterilized by 70% ethyl alcohol for 15 min. followed by 3 times rinsing with 1 ×phosphate buffer saline (PBS) for 30 min. L-929 cells (ATCC.CCL-1 NCTC clone 929) purchased from Biomedia, Thailand, (3 × 10^3^ Cells/Well), and culture medium were added into the wells and incubated at 37 °C for 3 days. Cell growth and appearance were observed under a microscope. The investigated nanofiber mats were stained with cell dye (crystal violet) and taken under the microscope to take pictures to count the increasing cells. The percentage of the cell adhesion on the fibrous membranes was calculated, while the cell formation was also considered successively.

### Cell cytotoxicity

Cell cytotoxicity of the fabricated nanofiber mats to cells was investigated by MTT assay adapted from Canadas et al., 2014^46^. Briefly, the fabricated nanofiber mats were loaded into 96-well plate. The nanofibers were pre-sterilized by 70% ethyl alcohol for 15 min. followed by 3 times rinsing with 1 ×phosphate buffer saline (PBS) for 30 min. L-929 cells (ATCC.CCL-1 NCTC clone 929) purchased from Biomedia, Thailand, (3 × 10^3^ Cells/Well), and culture medium were added into the wells and incubated at 37 °C for 3 days. Culture medium was aspirated out from the cultured plates. MEM-free serum and MTT reagent were added into the 96-well plates containing the nanofiber mats. The plates were incubated at 37 °C for 3 h. followed by adding the MTT solvent, and shaking for 15 min. Absorbance of each sample (B) was examined with UV–vis spectroscopy at 590 nm., and compared with the cell grownth on tissue culture plate as the control one (A). Cell cytotoxicity was considered from the percentage of the absorbance change computed by Eq. (3).

3 $$\% \; {\text{Cell Cytotoxicity}} = \left[ {\left( {{\text{A}} - {\text{B}}} \right)/{\text{A}}} \right] \times {1}00$$

## Results

Physical and surface characteristics, molecular structural property, wetting property, biodegradable property, tensile strength, cell adhesion and formation, and cell cytotoxicity were performed. All testing results are shown in Table 1 except the molecular structural outcome being reported separately.

**Table 1:** Nanofibers characteristics and property with different concentration ratio of SS and SF.

SS:SF (%w/v)	Diameter size (nm.)	Average contact angle (°)	Degradation at day 15 (%)	Young's modulus (MPa)	Cell adhesion (%)	Cell Cytotoxicity (%)
0.0:0.0	311.191 ± 112.012	0	0	412.680 ± 56.003	42.037 ± 12.096	28.989 ± 4.701
2.5:2.5	232.979 ± 57.877	0	1.961 ± 3.396	1,869.492 ± 318.709	50.185 ± 5.507	35.638 ± 5.265
3.0:2.0	241.771 ± 87.955	0	0	1,159.830 ± 326.057	49.629 ± 2.081	35.904 ± 0.188
3.5:1.5	249.594 ± 69.667	0	0.695 ± 1.202	924.211 ± 232.345	48.148 ± 11.547	35.979 ± 3.385
4.0:1.0	255.065 ± 62.155	0	0	787.798 ± 170.746	45.556 ± 13.892	32.181 ± 6.111
4.5:0.5	260.777 ± 95.073	0	0	666.694 ± 238.014	43.148 ± 10.692	32.477 ± 3.949
5.0:0.0	267.836 ± 96.419	0	0	456.369 ± 272.731	42.407 ± 8.144	33.245 ± 2.087

### Morphological structures

Physical and surface characteristics of the fabricated nanofibers were characterized by SEM (× 5000). SEM images were analyzed by ImageJ software to determine the average diameter size of the fibers.

**Figure 1:**
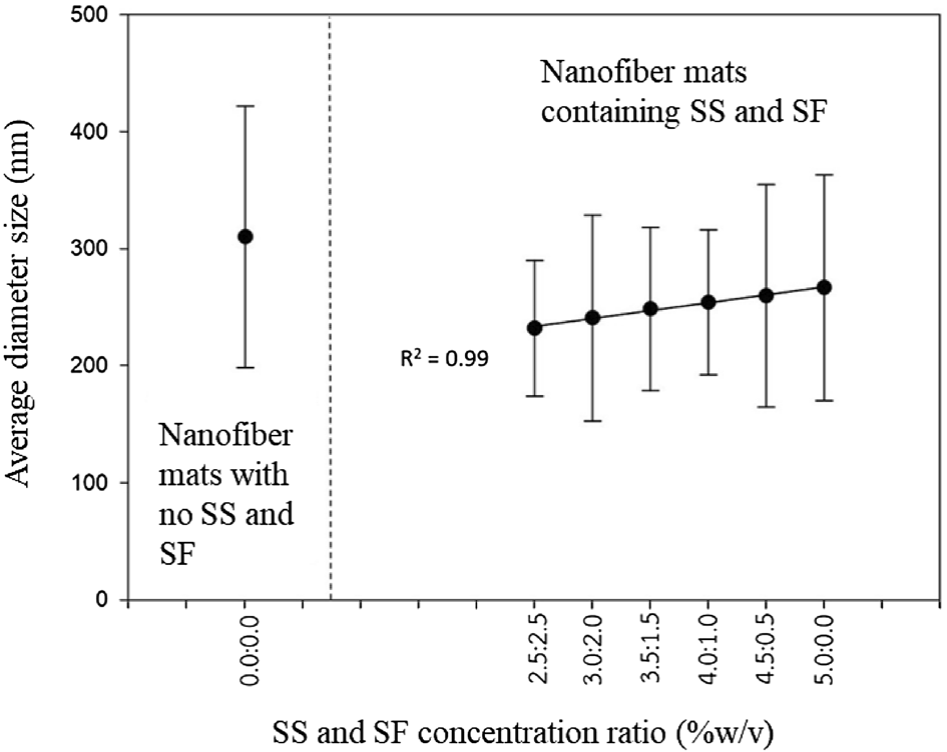
Average diameter size of the fabricated nanofibers with different SS/SF concentration ratio.

The average diameter size of PCL/CA nanofibers with different SS/SF concentration ratio are linearly correlated (R^2^ = 0.99) as seen in Fig. 1. The average diameter size of the fabricated fibers is directly proportional to the SS/SF concentration ratio, the diameter of the fabricated nanofibers increases when increasing the concentration of SS to SF. Size of the PCL/CA nanofibers that do not contain SS and SF is higher than that of the fibers containing SS and SF in all fabricating conditions.

**Figure 2:**
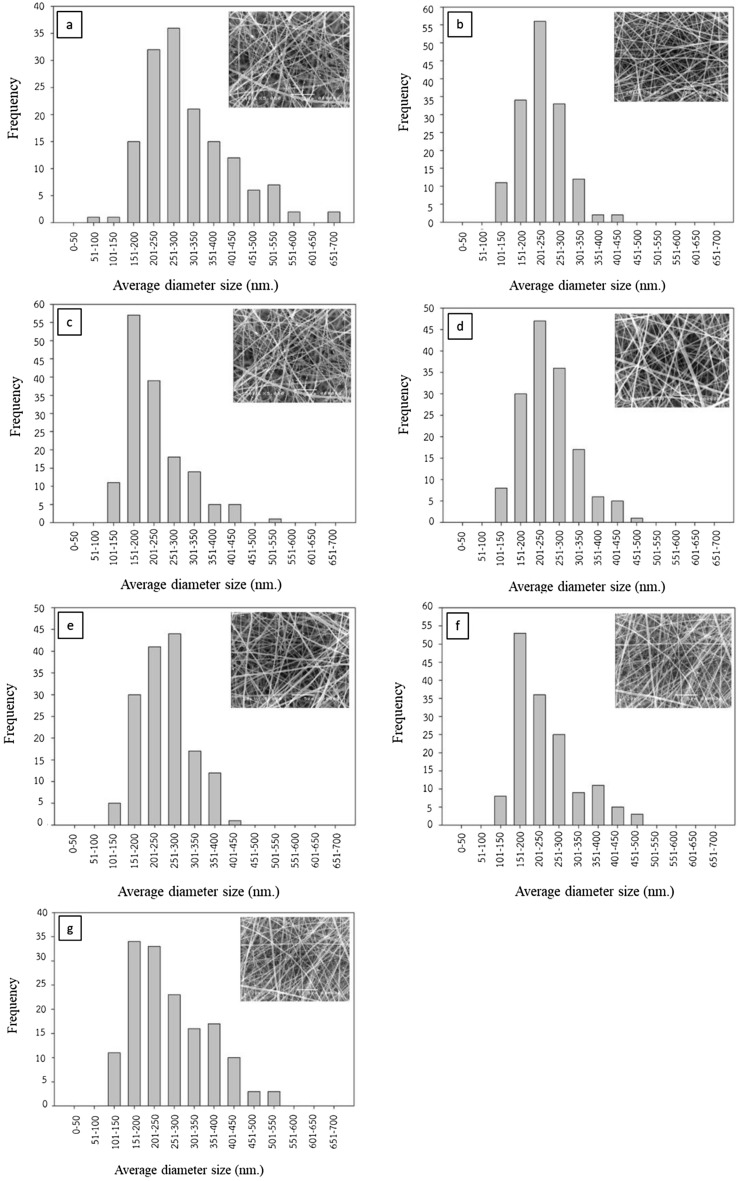
The distribution of the average diameter size of the fabricated nanofibers with SS/SF concentration ratio (%w/v) of (**a**) 0.0:0.0 (**b**) 2.5:2.5 (**c**) 3.0:2.0 (**d**) 3.5:1.5 (**e**) 4.0:1.0 (**f**) 4.5:0.5 (**g**) 5.0:0.0.

The distribution of the average diameter size of the fabricated nanofibers with SS/SF concentration ratio (%w/v) is shown in Fig. 2. Not only the average diameter size of the fibers, but also the nanofiber size distribution is affected by the SS/SF concentration ratio. It is clearly observed that the fiber diameter has a narrower distribution range when the fibers are fabricated by the polymer solution with both SS and SF than one without SS and SF or with only SS.

### Molecular structural property

**Figure 3:**
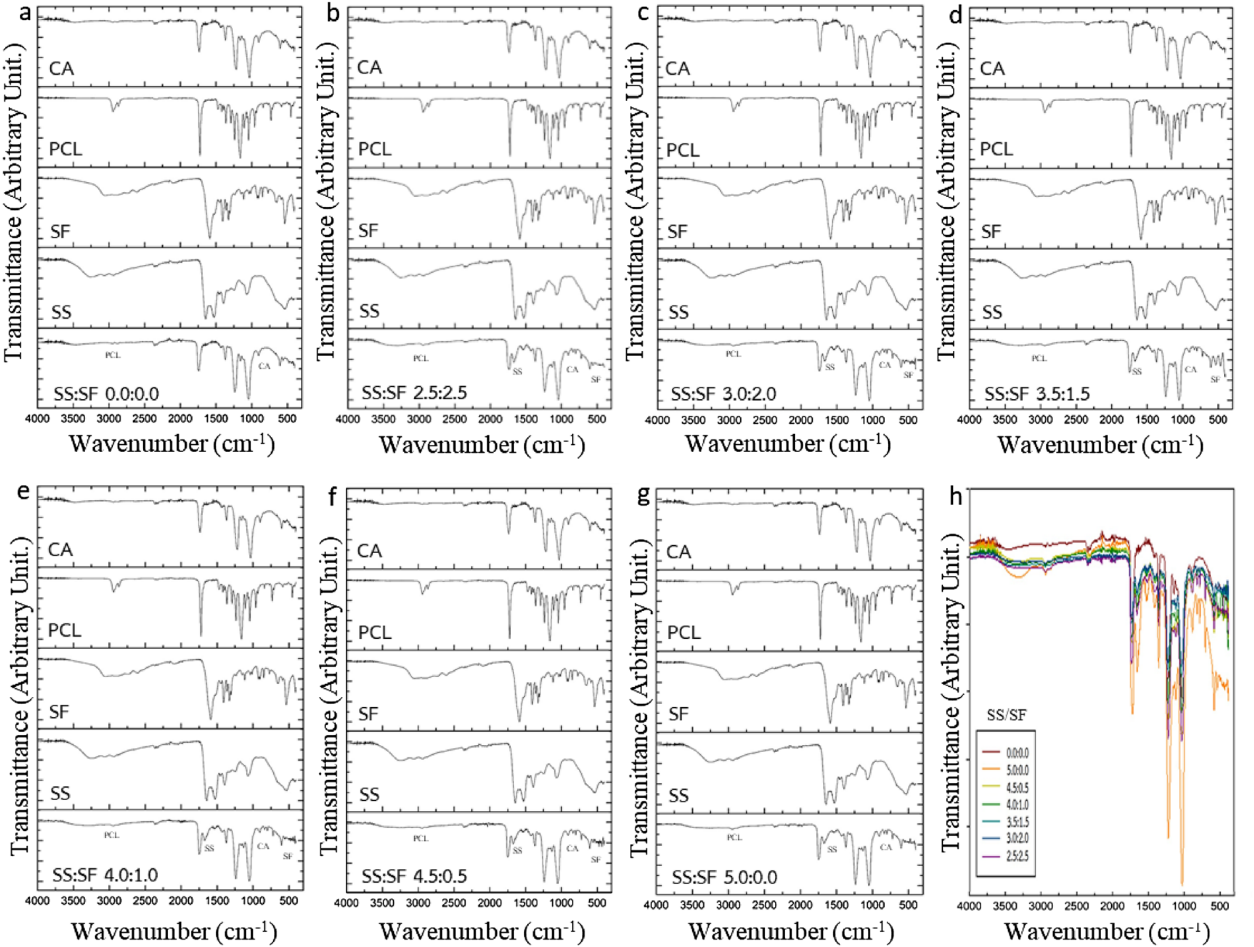
The Fourier-transform infrared (FTIR) spectra of the fabricated nanofiber mats with SS/SF concentration ratio of (**a**) 0.0:0.0 (**b**) 2.5:2.5 (**c**) 3.0:2.0 (**d**) 3.5:1.5 (**e**) 4.0:1.0 (**f**) 4.5:0.5 (**g**) 5.0:0.0 comparing to pure CA, PCL, SF, and SS, and (**h**) is the FTIR spectra comparing of nanofiber mats of all SS and SF concentration ratios.

Nanofiber mats fabricated from different concentration ratios of SS and SF were characterized by the Fourier-transform infrared spectroscopy (FTIR) to investigate their persistence of the substances used for nanofiber fabrication. The FTIR spectra of nanofiber mats containing SS and SF at different concentration ratio comparing to pure CA, PCL, SF, and SS, and the FTIR spectra comparing of nanofiber mats of all SS and SF concentration ratio are shown in Fig. 3 a–g and h, respectively. The FTIR spectrum results show strong absorption peak at 900, 2,935, 1,639 and 545 cm^−1^, indicating the chemical structure of the CA, PCL, SS, and SF derivative, respectively. The spectrum peaks of different concentration ratios of SF and SS are found at the positions of the wave number closed to pure SF and SS (Fig. 3h). It can be concluded that the SF and SS present in every nanofiber fabrication conditions with SF and SS in precursor solution.

### Wetting property

Wettability of fabricated nanofiber mats was investigated and interpreted by the contact angle measured by contact angle meter. The contact angles are determined by the angle between the water droplets and the surface of the nanofiber mats. Average contact angles of all prepared fiber mats are zero degrees listed in Table 1. This means that all obtained nanofibers are superhydrophilic.

### Biodegradable property

By the immersion technique, all fabricated nanofibers mats were soaked into the stimulated body fluid (SBF) solution for various timeframes up to 15 days. Percentage of degradation at day 15 for all samples are listed in Table 1. The persistence percentage of the fiber sheets tends to be stable over the test period of 100%, indicating that the biodegradation percentage of the fiber mats is likely to remain constant at 0%, significantly. Therefore, from the biodegradation test of the fiber sheets, it can be inferred that the fibrous membranes are able to maintain a shelf life of not less than 15 days, which is sufficient for cell adhesion of approximately 7 h, and sufficient for cell adhesion, growth, and division which takes approximately at least 3 days.

### Tensile Strength

**Figure 4:**
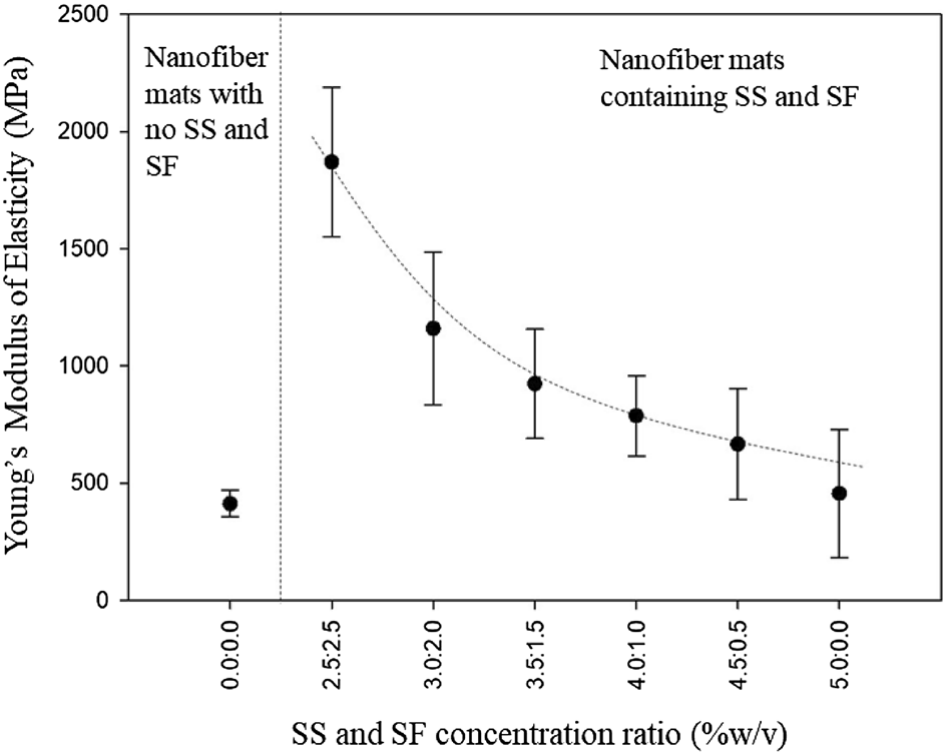
Young’s modulus of elasticity of the fabricated nanofiber mats with different SS/SF concentration ratio.

Stress and strain of all fabricated nanofiber mats were interpreted by the information obtained from the tensile strength testing. Young’s Modulus of elasticity of each fiber mat is computed from the slope of the linear range of the stress–strain relationship, and listed in Table 1. The Young’s Modulus of elasticity of all samples are plotted and compared in Fig. 4. Young's modulus of elasticity tends to decrease as the SS concentration in the fiber mat is increased (the proportion of the SF concentration in the fiber sheet is reduced). Moreover, the Young’s modulus of elasticity of nanofiber sheets without SS and SF is lower than that of SS/SF containing fiber sheets under all conditions of fabrication.

### Cell adhesion and formation

**Figure 5:**
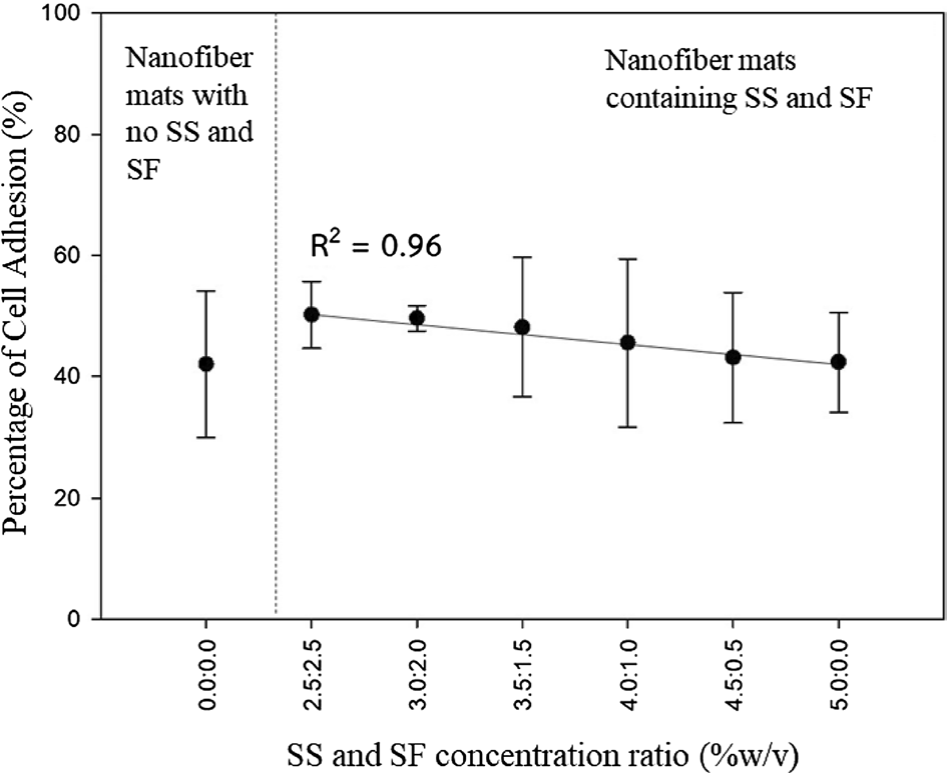
Cell adhesion on the fabricated nanofiber mats with different SS/SF concentration ratio.

Cell adhesion and formation were investigated together with the similar process by L-929 cell culturing on the fabricated nanofiber mats. The percentage of the cell adhesion on the fibrous membranes is calculated and reported in Table 1. The relationship of the percentage of the cell adhesion and the SS/SF concentration ratio for the fiber fabrication is shown in Fig. 5.

The percentage of the cell adhesion on PCL/CA nanofibers shows a linear relationship (R^2^ = 0.96) with the SS to SF concentration ratio. The percentage of the cell adhesion on the fabricated fiber sheet is inversely proportional to the increased percentage of SS over SF concentration. However, the percentage of the cell adhesion on the fiber sheets in each fabricated condition is not significantly different. It is noticed that the percentage of the cell adhesion on the PCL/CA nanofibers without SS and SF is lower than the percentage of the cell adhesion on the SS- and SF-containing fibers under all conditions of fabrication.

**Figure 6:**
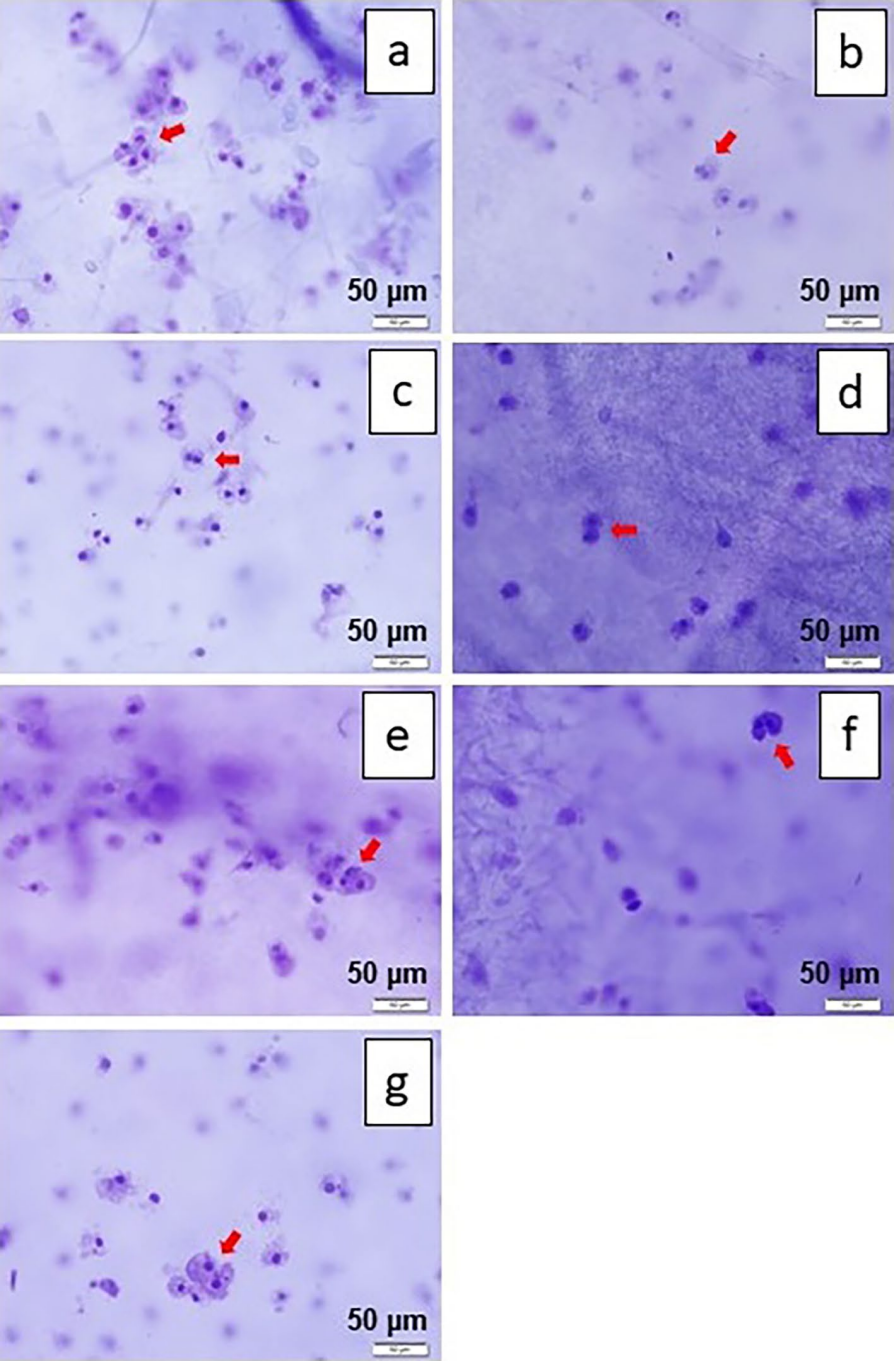
Cell formation and division on the fabricated nanofiber mats with SS/SF concentration (%w/v) ratio of (**a**) 0.0:0.0 (**b**) 2.5:2.5 (**c**) 3.0:2.0 (**d**) 3.5:1.5 (**e**) 4.0:1.0 (**f**) 4.5:0.5 (**g**) 5.0:0.0 with scale bar of 50 µm.

Cells on the fibrous membranes under all fabricating conditions tested can attach on the fiber mats and divide. This can be confirmed by considering the cell adhesion on the fiber sheets for all fabricating conditions. The division of the nucleus into new cells is shown in Fig. 6. It can be represented that cells are likely to grow and develop into new tissues on PCL/CA nanofibers fabricated both with and without SS and SF.

### Cell cytotoxicity

**Figure 7:**
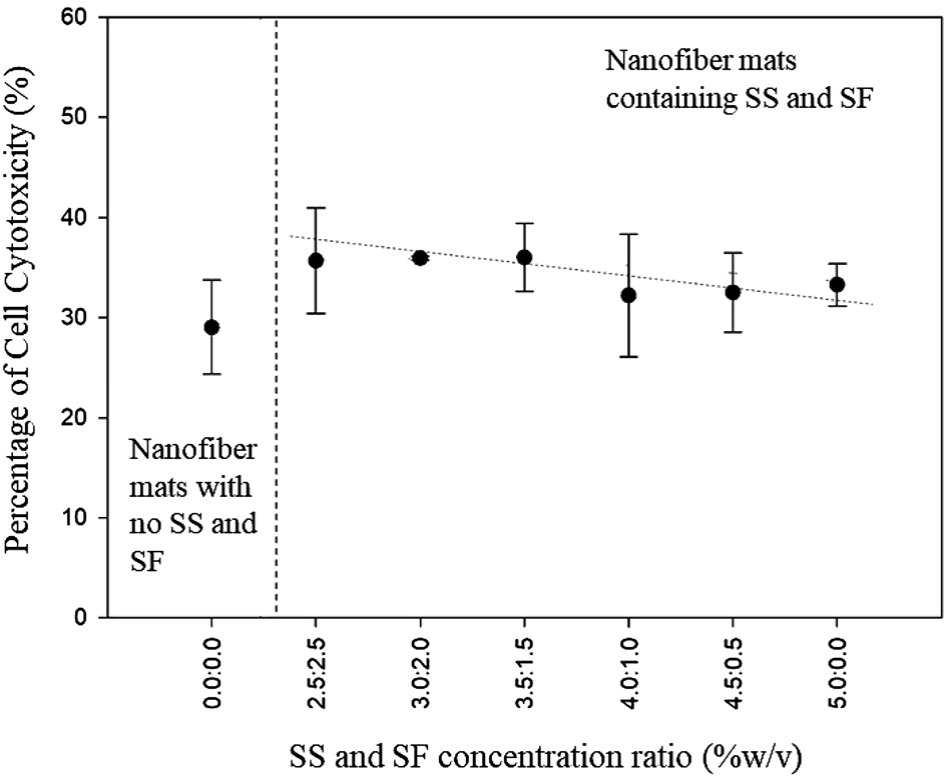
Cell cytotoxicity of the fabricated nanofiber mats with different SS/SF concentration ratio.

MTT assay was conducted to examine the cell cytotoxicity of the fabricated nanofiber mats. The percentage of cell cytotoxicity is listed in Table 1. The linear relationship between the percentage of the cell cytotoxicity and the SS to SF concentration ratio is illustrated in Fig. 7. The fabricated fiber sheets under all conditions have a low percentage of cell cytotoxicity with 29–36%, approximately. The percentage of the cell cytotoxicity of fibers tends to decrease with the increasing of SS to SF concentration ratio. However, the cell cytotoxicity percentages of these fibers are not significantly different. The cell cytotoxicity percentages of SS-and SF-containing fibers are slightly higher than that of non-SS- and SF-containing fibers.

## Discussion

Different concentration ratio of SS and SF contained in nanofiber mats, primary polymers (PCL and CA), and also the solvent (TFA) used for fiber fabrication affect not only the physical and surface characteristics, but also the initial medical property of the fibrous membranes. The diameter of the fabricated nanofibers increases when increasing the concentration of SS to SF concentration ratio which can be described that the slurry becomes more viscous when the amount of SS increases, resulting in greater fiber diameter size^47,48^. The obtained SS/SF containing fiber sizes are in between 201 and 300 nm, which also has a smaller size and narrower range of the fiber size dispersion comparing to non-SS-SF-containing fibers. The FTIR peaks of wave number positions of the fiber sheets with different concentrations of SS and SF are similar. The dominant peaks are found at the positions of wave numbers approximately 900, 2,935, 1,639 and 545 cm^−1^, indicating the structural positions of the CA, PCL, SS, and SF derivative, respectively. PCL/CA nanofiber sheets that do not contain SS and SF, the peaks are not found at the wave number positions of approximately 1639 and 545 cm^−1^, which are the peaks indicating the locations of SS and SF derivatives. Superb wetting property of the fiber surface may occur due to the TFA, which is used as the solvent in the fiber fabrication process. TFA has a strong acidic effect resulting in the destruction of the bonding structure of CA, the main polymer, proceeding in the formation of a hydroxyl group (-OH), which has increased hydrophilicity. Consequently, the surface of the fabricated fiber mats evinces great hydrophilicity. Fabricated fibers can be maintained for a period at least 15 days with no significant degradation which is much greater comparing to the SS/SF nanofiber previous studied and fabricated by Zhang, et al.^40^ that easily dissolved in water within only 1 h. This is beneficial for cell growth, adhesion, and dividing into more stable and larger cells which is a good preliminary feature in medicine especially for cell scaffolding applications that required at least 1–3 days for cell attachment^36,39^. The great tested results in biodegradability may be due to the relatively long degradation time of the main polymers used in the fabrication of fiber mats, PCL, and CA, of more than 24 and 2 months, respectively. SS and SF degradation time of more than 2 months might also help to prolong the degradation period^49,50^. The nanofiber sheets with and without SS and SF fabricated in this study demonstrate the Young’s modulus of elasticity in the range of 412.680 MPa and 1869.492 MPa which close to that of bones^36^, whereas the elastic modulus of the PCL nanofibers and SS/SF nanofibers previously observed by Baker, et al.^37^ and Hang, et al.^38^ are only at 60 MPa and 5.5 MPa, respectively. The fabricated nanofibers with SS/SF in this work with greater modulus are suitable for scaffold development especially for bone regeneration due to a reason that an ideal scaffold must have similar mechanical properties to the transplanted organ^35^. The Young's modulus of elasticity tends to decrease as the SS concentration in the fiber mats is increased. This may be due to the properties of SS that contribute to the strength of the materials^51–53^, that is, the fiber mats have more hardness when the SS concentration used in the fabrication increases affecting in a decrease in flexibility. Cell adhesion percentages of the nanofibers with and without a mixture of SS and SF range from 42.037 to 50.185%. The adhesion of cells on nanofibers is found to be greater for the fabricated nanofibers containing SS and SF. The smaller fiber diameter found when the SS content in the fabricated solution is reduced, directly results in the greater cell adhesion. This may be due to the characteristic of the nanofibers, an increase in surface area to volume ratio^50,54–56^. It is also found that the cells on the fibrous membrane under each condition of fabrication form and divide the nucleus to form new cells and tend to grow and develop into new tissues. However, the change in the SS/SF concentration ratio under this study insignificantly affects the cell cytotoxicity. The percentages of cell cytotoxicity of the nanofibers with and without a mixture of SS and SF are low in the range of 28.989–35.979%. The average percentage of the cell cytotoxicity is less than 50% indicating that the fiber mats are not toxic to cells^57^. Among their positive outcomes in each investigation, the fabricated fibers with SS and SF ratio of 2.5:2.5 (% w/v) are the most promising and appropriate for further biomedical development.

## Conclusions

In this research, polycaprolactone/cellulose acetate nanofibers containing a mixture of sericin and fibroin were successfully fabricated by electrostatic spinning process to study the effect of sericin and fibroin concentrations on the size and properties of fabricated nanofibers for biomedical application. The size of the nanofibers containing sericin and fibroin is increased when the concentration percentage of sericin used in the fabrication process is increased (the percentage of fibroin concentration is decreased). The composition of the fabricated nanofibers was verified by the FTIR investigation. All fabricated fiber sheets show superhydrophilic properties. The nanofiber sheets with, and without sericin and fibroin exhibit slow biodegradation which is sufficient and suitable for cell adhesion and regeneration. The elastic modulus of the fiber sheet is decreased when increasing the concentration of sericin in the fabrication process (reducing the percentage of fibroin concentration). The nanofiber sheets that do not contain sericin and fibroin show lower elastic modulus than that of sericin- and fibroin-containing fiber sheets under all conditions of fabrication. All fabricated nanofibers provide excellent mechanical property required for cell development in tissue engineering application. The cell adhesion is inversely proportional to the increased sericin concentration ratio (decreasing in fibroin concentration ratio). However, the percentage of cell adhesion of the fabricated fiber sheets in each condition is not significantly different. The percentage of cell adhesion on the nanofibers that do not contain sericin and fibroin is lower than that of the sericin- and fibroin-containing fibers under all conditions of fabrication. The percentage of cell cytotoxicity of the nanofibers with and without a mixture of sericin and fibroin tends to decrease as the sericin to fibroin concentration ratio is increased.

In summary, it can be concluded that the polycaprolactone/cellulose acetate nanofibers containing sericin and fibroin that are conveniently fabricated by electrostatic fiber spinning process show great initial biomedical requirement for scaffold development, especially influencing in much higher elasticity with slow degradation, and promoting more cell adhesion. These fabricated nanofibers are suitably used in biomedical application such as a cell scaffold, which could help in reducing expensive medical equipment and promoting novel low-cost tissue engineering materials.

## Data Availability

The datasets generated during and/or analyzed during the current study are available from the corresponding author on reasonable request.
